# A Single Dual-Function Enzyme Controls the Production of Inflammatory NOD Agonist Peptidoglycan Fragments by *Neisseria gonorrhoeae*

**DOI:** 10.1128/mBio.01464-17

**Published:** 2017-10-17

**Authors:** Jonathan D. Lenz, Kathleen T. Hackett, Joseph P. Dillard

**Affiliations:** Department of Medical Microbiology and Immunology, University of Wisconsin—Madison, Madison, Wisconsin, USA; Fred Hutchinson Cancer Research Center

**Keywords:** *Neisseria gonorrhoeae*, high-pressure liquid chromatography, innate immunity, peptidoglycan, peptidoglycan hydrolases

## Abstract

*Neisseria gonorrhoeae* gonococcus (GC) is a Gram-negative betaproteobacterium and causative agent of the sexually transmitted infection gonorrhea. During growth, GC releases lipooligosaccharide (LOS) and peptidoglycan (PG) fragments, which contribute significantly to the inflammatory damage observed during human infection. In ascending infection of human Fallopian tubes, inflammation leads to increased risk of ectopic pregnancy, pelvic inflammatory disease, and sterility. Of the PG fragments released by GC, most are disaccharide peptide monomers, and of those, 80% have tripeptide stems despite the observation that tetrapeptide stems make up 80% of the assembled cell wall. We identified a serine-protease l,d-carboxypeptidase, NGO1274 (LdcA), as the enzyme responsible for converting cell wall tetrapeptide-stem PG to released tripeptide-stem PG. Unlike characterized cytoplasmic LdcA homologs in gammaproteobacteria, LdcA in GC is exported to the periplasm, and its localization is critical for its activity in modifying PG fragments for release. Distinct among other characterized l,d-carboxypeptidases, LdcA from GC is also capable of catalyzing the cleavage of specific peptide cross-bridges (endopeptidase activity). To define the role of *ldcA* in pathogenesis, we demonstrate that *ldcA* disruption results in both loss of NOD1-dependent NF-κB activation and decreased NOD2-dependent NF-κB activation while not affecting Toll-like receptor (TLR) agonist release. Since the human intracellular peptidoglycan receptor NOD1 (hNOD1) specifically recognizes PG fragments with a terminal *meso*-DAP rather than d-alanine, we conclude that LdcA is required for GC to provoke NOD1-dependent responses in cells of the human host.

## IMPORTANCE

The macromolecular meshwork of peptidoglycan serves essential functions in determining bacterial cell shape, protecting against osmotic lysis, and defending cells from external assaults. The conserved peptidoglycan structure, however, is also recognized by eukaryotic pattern recognition receptors, which can trigger immune responses against bacteria. Many bacteria can induce an inflammatory response through the intracellular peptidoglycan receptor NOD1, but *Neisseria gonorrhoeae* serves as an extreme example, releasing fragments of peptidoglycan into the environment during growth that specifically antagonize human NOD1. Understanding the peptidoglycan breakdown mechanisms that allow *Neisseria* to promote NOD1 activation, rather than avoiding or suppressing immune detection, is critical to understanding the pathogenesis of this increasingly drug-resistant organism. We identify a peptidoglycan l,d-carboxypeptidase responsible for converting liberated peptidoglycan fragments into the human NOD1 agonist and find that the same enzyme has endopeptidase activity on certain peptidoglycan cross-links, the first described combination of those two activities in a single enzyme.

## INTRODUCTION

Peptidoglycan (PG) is a critical structural component of both Gram-positive and Gram-negative bacteria and is arrayed outside the cytoplasmic membrane, where it provides protection from osmotic stress and environmental insult. While Gram-positive bacteria have a thick PG layer on their surface, Gram-negative bacteria possess a much thinner PG layer that is situated between the inner (cytoplasmic) and outer membranes. The intact peptidoglycan layer (sacculus) is composed of glycan strands with repeating units of *N*-acetylglucosamine (GlcNAc) and *N*-acetylmuramic acid (MurNAc), cross-linked with short peptide chains containing noncanonical d-amino acids. During growth and cell division of Gram-negative bacteria, new PG must be inserted into the peptidoglycan cell wall, requiring localized breakdown of the PG matrix to accommodate the new material. The PG fragments generated during growth can be taken up by the cell and recycled into the precursor molecules used for PG synthesis or broken down for use in general metabolism (1). During PG recycling in *Escherichia coli*, fragments reenter the cytoplasm via the inner membrane permease AmpG or the oligopeptide permease OppA. Once in the cytoplasm, fragments are processed by an *N*-acetylglucosaminidase (NagZ) which separates GlcNAc from MurNAc, an amidase (AmpD) which separates the sugar moiety from the peptide stem, and peptidases that trim amino acids from the peptide stem (2, 3). In order to reuse fragments for PG synthesis, any PG fragment with an l-Ala-γ-d-Glu-*meso*-DAP-d-Ala (tetrapeptide) stem must first be processed to an l-Ala-γ-d-Glu-*meso*-DAP (tripeptide) stem to accept the dipeptide d-Ala-d-Ala from the peptide ligase MurF as part of regenerating the precursor UDP-MurNAc-pentapeptide (4). The reaction that separates a terminal d-alanine from *meso*-diaminopimelic acid (*m*DAP) during recycling is performed by the use of an l,d-carboxypeptidase, so named for the l-d bond between the l center of *m*DAP and d-Ala (5).

Peptidoglycan l,d-carboxypeptidases have been identified across Gram-positive and Gram-negative bacteria, with several different classes of enzymes able to accomplish the same activity. Enzymes with a serine-protease active site have been crystalized from the gammaproteobacterium *Pseudomonas aeruginosa* and the alphaproteobacterium *Novosphingobium* (*Sphingomonas*) *aromaticivorans* (5, 6). The best characterized of the serine protease l,d-carboxypeptidases is LdcA from *E. coli*, which functions in the cytoplasm to process non-cross-linked tetrapeptide stems to tripeptide stems. Deletion of *ldcA* from *E. coli* results in a buildup of UDP-MurNAc-tetrapeptide (considered a dead-end intermediate of PG biosynthesis) and lysis of cells as they reach stationary phase (7, 8). The epsilonproteobacteria *Helicobacter pylori* and *Campylobacter jejuni* each contain l,d-carboxypeptidases (*csd6* and *pgp2*, respectively) which contribute to the maintenance of helical shape, a property important for the motility and pathogenesis of spiral-shaped bacteria (9, 10). Csd6 has been crystallized and found to be structurally unrelated to *E. coli* LdcA, utilizing a His/Gly/Cys catalytic triad more closely related to l,d-transpeptidases than other l,d-carboxypeptidases (but possessing no l,d-transpeptidase activity) (11). In Gram-positive bacteria, the l,d-carboxypeptidase DacB was functionally characterized in both *Streptococcus pneumoniae* (12) and *Lactococcus lactis* (13) before versions were crystalized from *S. pneumoniae* and two *Bacillus* strains (14, 15). DacB was subsequently renamed LdcB to better reflect its function as an l,d-carboxypeptidase, though it occupies another class of enzyme different from those previously described: zinc-dependent metallopeptidases of the LAS (lysostaphin, d-Ala-d-Ala metallopeptidases, sonic hedgehog) family. Different bacteria have evidently found different ways to achieve l,d-carboxypeptidase activity, and the consequences of l,d-carboxypeptidase deletion on sacculus composition are equally different, ranging from minor alterations in the PG cross-linking profile (8) to total ablation of tripeptide PG stems from the sacculus (9).

Being produced within the periplasmic space, PG fragments from Gram-negative bacteria can typically be efficiently recycled without escaping the cell. Released PG fragments can become a liability for bacteria when they are detected by the host immune system via the intracellular PG sensors NOD1 and NOD2, which trigger inflammatory responses (16, 17). While the process of efficient PG breakdown and recycling may be viewed as a strategy to both minimize energy waste and escape immune detection, there are Gram-negative bacteria for which less-efficient PG recycling is the norm and released inflammatory PG fragments are a part of their interaction with the host. The best-known example is *Bordetella pertussis*, which releases tracheal cytotoxin (TCT), a PG fragment with the composition of *N*-acetylglucosaminyl-1,6-anhydromuramyl-tetrapeptide (tetrapeptide monomer) (18). Exposure to TCT causes inflammation and leads to the death of ciliated cells in the respiratory epithelium (19). The squid symbiont *Vibrio fischeri* also uses a PG fragment structurally identical to TCT to manipulate its host during the process of mutualistic colonization (20). *Neisseria gonorrhoeae* gonococcus (GC) releases soluble anhydro-muramyl PG fragments during growth, totaling about 15% of the PG fragments liberated from the cell wall during each division cycle, compared to about 3% to 5% from *E. coli* (21, 22). During ascending *N. gonorrhoeae* infection of the female reproductive tract, Fallopian tube ciliated epithelial cells are killed in a manner reminiscent of the damage caused by TCT. It was previously demonstrated that the loss of ciliated cells during GC infection can be recapitulated by cell-free supernatant and, indeed, by purified PG monomers alone (23).

One unique and curious aspect of the inflammatory PG monomer fragments released by gonococci is that only about 20% represent the tetrapeptide monomer (identical to TCT) whereas 80% represent the smaller tripeptide monomer (terminating in *m*DAP) (24). While NOD1 has been shown to recognize PG fragments containing *m*DAP ranging from a tripeptide monomer down to a minimal dipeptide (d-Glu-*m*DAP, iE-DAP) (25), there is a critical difference between the murine version of NOD1 (mNOD1), which preferentially responds to tetrapeptide monomer (TCT), and the human version (hNOD1), which responds instead to tripeptide monomer (and responds poorly to TCT) (26). The single amino acid change in the length of the released PG peptide stem therefore has major implications for the innate immune response to GC, for which humans are the single host species. The release of primarily tripeptide-stem PG fragments by GC is also notable considering that the assembled sacculus of GC is composed of 80% tetrapeptide stems (27), implying that a mechanism exists to convert the tetrapeptide stems present in the periplasmic sacculus to tripeptide stems prior to any cytoplasmic recycling activity.

In this work, we describe the identification and characterization of a peptidoglycan l,d-carboxypeptidase (NGO1274 [LdcA]) from GC that addresses the issue of how tripeptide PG fragments are produced by this bacterium. We show that, unlike other characterized Gram-negative serine-protease LdcA homologs, GC LdcA is exported to the periplasm, where it associates with the outer membrane. By analyzing both the profile of released PG fragments and the composition of assembled sacculi, we determined that the localization of this enzyme is critical for its function in PG fragment release and recycling. Mutations that (i) disable the active site of LdcA, (ii) restrict LdcA to the cytoplasm, or (iii) eliminate LdcA entirely all have identical phenotypes: an ablation of released tripeptide, loss of free tripeptide stems in the sacculus, and a loss of the ability of GC to activate inflammatory responses through hNOD1. Using reporter cells for hNOD1-dependent NF-κB activation, we identify LdcA as the sole enzyme in GC responsible for modifying liberated PG fragments into hNOD1 agonists, with an additional effect on the generation of hNOD2 agonist, likely through decreased supply of tripeptide substrate to make dipeptide. Unexpectedly, disruption of normal LdcA activity results in a much higher than normal release of PG fragments with monomer units still linked by intact peptide cross-bridges (referred to as peptide-linked dimers). We found that, when provided with peptide-linked dimers as the substrate, LdcA from GC can process these PG fragments to tripeptide monomers, using a combination of carboxypeptidase and endopeptidase activities not previously described for serine protease l,d-carboxypeptidases or related classes of enzymes.

## RESULTS

### NGO1274 encodes a periplasmic peptidoglycan l,d-carboxypeptidase (LdcA).

**FIG 1:**
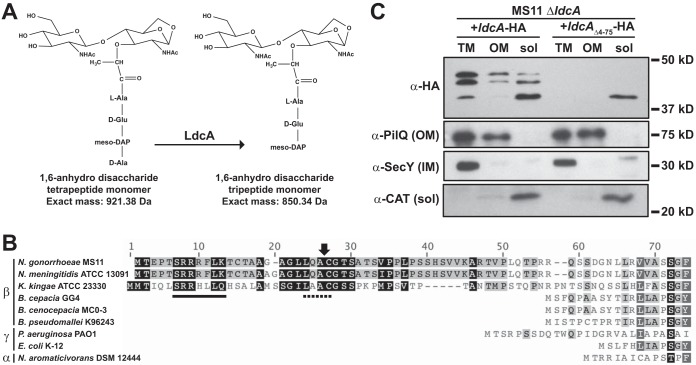
LdcA is exported from the cytoplasm in *N. gonorrhoeae* gonococci (GC). (A) l,d-Carboxypepdidases catalyze the removal of the terminal d-alanine from PG peptide stems, leaving *m*DAP as the terminal amino acid. (B) ClustalW alignment of serine protease LdcA sequences from alphaproteobacteria, betaproteobacteria, and gammaproteobacteria. The sequences were obtained from NCBI. The solid underline indicates a consensus TAT motif, the dotted line indicates a lipobox motif, and the arrow indicates the location of the predicted signal peptidase cleavage site. *K. kingae*, *Kingella kingae*; *B. cepacia*, *Burkholderia cepacia*; *B. cenocepacia*, *Burkholderia cenocepacia*; *B. pseudomallei*, *Burkholderia pseudomallei*. (C) Subcellular fractionation and Western blot analysis of GC strain MS11 without native *ldcA*, expressing C-terminally HA-tagged versions of either full-length *ldcA* or *ldcA* with a deletion of the N-terminal leader sequence (*ldcA*_Δ4−75_). Total membrane (TM), outer membrane (OM), and soluble (sol) fractions are indicated. The soluble fraction includes both periplasm and cytoplasm. All samples were probed with control antibodies for proteins located in the outer membrane (PilQ), inner membrane (SecY), and cytoplasm (chloramphenicol acetyltransferase [CAT]; expressed from an integrated plasmid).

The assembled peptidoglycan (PG) sacculus of *N. gonorrhoeae* (GC) is composed of a higher proportion of disaccharide units bearing tetrapeptide stems (80%) than of those bearing tripeptide stems (20%) (with a minor share of those bearing pentapeptide and dipeptide stems) (27). The soluble fragments of PG released during GC growth, however, represent nearly opposite proportions. PG monomers (GlcNAc-anhMurNAc-peptide fragments) account for the largest share of released fragments, with 80% released as GlcNAc-anhMurNAc-tripeptide (GaM-3) and 20% as GlcNAc-anhMurNAc-tetrapeptide (GaM-4) (24). To account for the observed difference in released fragments, we hypothesized that an l,d-carboxypeptidease removes the terminal d-alanine from PG fragments produced during the turnover of sacculi by lytic transglycosylases (glycan-processing enzymes that liberate PG fragments and produce the 1,6-anhydro bond on released fragments) (Fig. 1A). Using the sequence of the peptidoglycan l,d-carboxypeptidase *ldcA* from *E. coli* in a BLAST search of the *N. gonorrhoeae* strain MS11 genome, we identified NGO1274 as a serine protease in the peptidase_S66 family and a putative l,d-carboxypeptidase. The 1,182-bp gene encodes a 394-amino-acid protein that is somewhat longer than the *E. coli* K-12 homolog and yet shares 52.7% identity with it (Fig. 1B). Alignment data suggest the presence of the same serine-histidine-glutamic acid catalytic triad residues as those predicted from the *Pseudomonas aeruginosa* LdcA crystal structure (Ser165, Glu260, and His331 in GC) (5).

A notable feature of GC *ldcA* is the addition of >150 bp at the 5′ end compared to *ldcA* from the gammaproteobacteria *E. coli* and *P. aeruginosa* (Fig. 1B). The additional sequence is not a common feature of all betaproteobacteria (*Burkholderia* spp. do not have additional sequence compared to *E. coli*) but is a common feature of the *Neisseriaceae*, including both pathogenic and commensal *Neisseria* as well as *Kingella* (Fig. 1B; see also Fig. S1 in the supplemental material). Included in this N-terminal extension is the sequence SRRXXL, which indicates a twin-arginine-translocase (TAT)-dependent signal peptide. Analysis of the N-terminal end of LdcA using the TatP 1.0 server predicts a TAT-dependent signal peptide and a potential cleavage site between residues 29 and 30 (28). No Sec-dependent signal peptide is predicted by SignalP 4.1 (29).

10.1128/mBio.01464-17.1 FIG S1  LdcA is conserved throughout pathogenic and commensal *Neisseria* gonococci and across host species. Results of ClustalW alignment of LdcA sequences from selected human- and animal-associated *Neisseria* strains are shown. Asterisks indicate active site residues. The sequences were obtained from NCBI. Download FIG S1, EPS file, 2.8 MB. Copyright © 2017 Lenz et al. 2017 Lenz et al. This content is distributed under the terms of the Creative Commons Attribution 4.0 International license.

To determine where LdcA localizes in *N. gonorrhoeae*, two versions of *ldcA* were cloned into GC complementation vector pMR68 (30): one full-length version of *ldcA* and one truncated version lacking nucleotides 4 to 75 (*ldcA*_Δ4−75_). Both versions retain the native ribosome-binding site and native start codon and have added sequence at the 3′ end to generate a C-terminal hemagglutinin (HA) tag. Expression of each construct was induced (with anhydrotetracycline [aTc]) from the chromosome of a GC strain with a complete in-frame deletion of native *ldcA*. Bacteria expressing tagged *ldcA* were then subjected to subcellular fractionation to produce outer membrane (OM), total membrane (TM, inner and outer), and soluble (Sol, cytoplasm and periplasm) fractions. Fractions were subjected to SDS-PAGE analysis and Western blotting for the HA tag to localize LdcA-HA and LdcA_Δ2−25_-HA (Fig. 1C). Identical samples were analyzed on separate gels using antibody against PilQ (OM control), SecY (IM control), and chloramphenicol acetyltransferase (CAT) on the integrated pMR58 plasmid (cytoplasmic control). Fractionation controls indicated the expected enrichment of intended fractions.

Wild-type (WT) LdcA, which is predicted to be 42.3 kDa, was observed as three bands, all between 40 and 45 kDa. The presence of the two largest bands in both the soluble and outer membrane-associated fractions indicates that LdcA is capable of being exported from the cytoplasm, where it associates with the outer membrane. The localization pattern observed for the larger LdcA forms is the same as that observed for the known outer membrane protein PilQ and different from that observed for the known inner membrane protein SecY, indicating that LdcA associates with the outer membrane. The two larger bands present in the wild-type soluble fraction are therefore likely located in the periplasm. When LdcA is truncated by removal of the signal sequence, only one band (likely representing the cytoplasm) is observed in the soluble fraction, where it does not appear to associate with any membranes. The presence of a small band of approximately the same size as that seen with *ldcA*_Δ4−75_ in the wild type, some of which associates with the inner membrane (in the TM but not the OM fraction), may represent the protein prior to signal-sequence processing though it is not clear if this smallest isoform is cytoplasmic or periplasmic.

### LdcA controls the production of soluble GlcNAc-anhydro-MurNAc-tripeptide monomer from *N. gonorrhoeae*.

**FIG 2:**
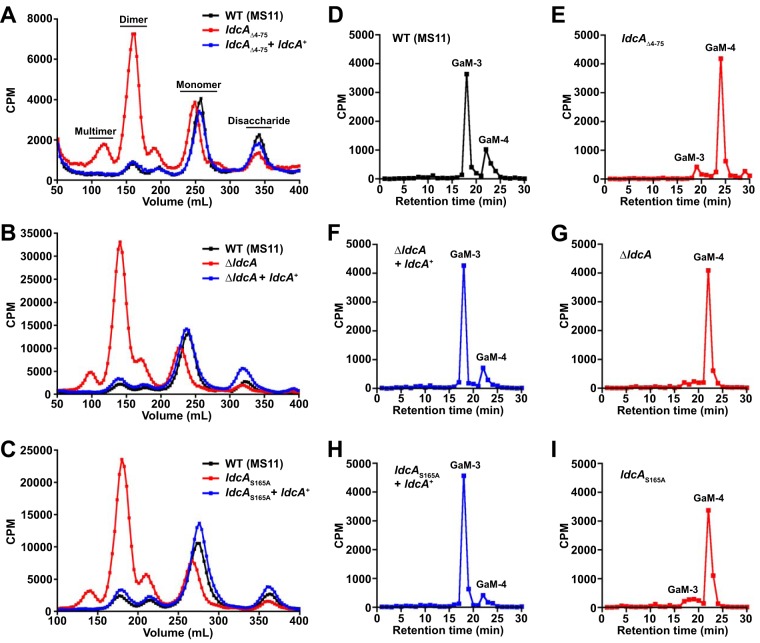
Disruption of LdcA activity alters peptidoglycan fragment release. (A to C) GC strains with a signal peptide truncation (*ldcA*_Δ4−75_), an in-frame deletion (Δ*ldcA*), or an active site mutation (*ldcA*_S165A_) were metabolically pulse-chase labeled with [^3^H]glucosamine alongside wild-type strain MS11 (WT) and the appropriate complementation strain (+*ldcA*^+^) in separate experiments. PG fragment release profiles were obtained by fractionating cell-free supernatant by size exclusion chromatography and measuring radiolabeling levels by liquid scintillation counting. Profiles for each set of three strains are representative of results from at least two independent growth, labeling, and size exclusion chromatography analyses. (D to I) PG monomers were obtained from size exclusion chromatography fractions, and data corresponding to 10,000 cpm were separated by reversed-phase HPLC on a C_18_ column. One-minute fractions were collected and measured by liquid scintillation counting. GaM-3, GlcNAc-anhydro-MurNAc-tripeptide monomer; GaM-4, GlcNAc–anhydro-MurNAc–tetrapeptide monomer.

Distinguishing features of *N. gonorrhoeae* are the inefficiency with which it recycles PG fragments and how many of the monomeric PG fragments are released during growth (22, 31). The released fragments bear the 1,6-anhydro bond characteristic of the products of lytic transglycosylases, and the lytic transglycosylase LtgD is responsible for generating most of the fragments destined for release (24, 32). What remained unclear was how a large proportion of the peptide stems of released PG monomer fragments become tripeptide (l-Ala-γ-d-Glu-*m*DAP) (24) while most stems in the sacculi are tetrapeptide (l-Ala-γ-d-Glu–*m*DAP–d-Ala) (27). To determine if *ldcA* is able to impact the profile of released PG fragments, we employed metabolic pulse-chase labeling of GC peptidoglycan followed by size exclusion chromatography to quantitatively measure the release of PG fragments from strains with three different alterations of *ldcA* as follows: (i) truncation of the signal sequence (identical to that used in the localization studies) (*ldcA*_Δ4−75_) (Fig. 2A), (ii) in-frame deletion (Δ*ldcA*) (Fig. 2B), and (iii) mutation in the putative serine protease active site (*ldcA*_S165A_) (Fig. 2C). Each of these mutants has the same alteration in the profile of released PG fragments; the bacteria release larger PG monomers (eluting earlier on size exclusion), and the amount of multimeric PG released is greatly increased. Mutations in other putative active site residues revealed a similar PG fragment release pattern for H331A (slightly more severe) and E260A (somewhat less severe) (Fig. S2A), indicating the presence of the same serine protease catalytic triad as that identified in *P. aeruginosa* LdcA (5).

10.1128/mBio.01464-17.2 FIG S2  Disruption of catalytic triad alters peptidoglycan fragment release. (A) GC strains with mutations in catalytic triad residues (*ldcA*_E260A_, *ldcA*_H331A_) were metabolically pulse-chase labeled with [^3^H]glucosamine alongside the strains represented in Fig. 2B. PG fragment release profiles were obtained by fractionating cell-free supernatant by size exclusion chromatography and measuring radiolabeling by liquid scintillation counting. (B and C) Radiolabeled PG monomers were obtained from size exclusion chromatography fractions, and results corresponding to 10,000 cpm were separated by reversed-phase HPLC. One-minute fractions were collected and measured by liquid scintillation counting. Download FIG S2, EPS file, 0.9 MB. Copyright © 2017 Lenz et al. 2017 Lenz et al. This content is distributed under the terms of the Creative Commons Attribution 4.0 International license.

Since PG monomers have demonstrated functions as inflammatory immune agonists in host-pathogen interactions, we were interested in characterizing changes in the amounts or types of released PG monomer fragments from *ldcA* mutants. PG monomers were harvested from fractions collected during size exclusion chromatography and subjected to analysis by reversed-phase high-pressure liquid chromatography (rpHPLC). The wild-type distribution of PG monomer was mostly GaM-3 with some GaM-4 (Fig. 2D). In the *ldcA*_Δ4−75_ mutant, however, the proportion of released monomer shifted to almost completely GaM-4 (Fig. 2E). Similar distributions of monomer were observed for each of the other *ldcA* mutant backgrounds, and overexpressing *ldcA* from a complementation site at a distant locus on the chromosome restored GaM-3 release (Fig. 2E to I and S2B and C). These results show that the l,d-carboxypeptidase activity that was long predicted to occur in GC to liberate tripeptide-stem PG (24) is the result of the presence of the protein that we have identified as LdcA. While it makes intuitive sense that the localization of this enzyme outside the cytoplasmic membrane would be critical for its activity with regard to released fragments, activity in this location represents a departure from that seen with the Gram-negative bacteria that use LdcA exclusively in cytoplasmic PG recycling. This is the only example reported to date of a Gram-negative l,d-carboxypeptidase altering the profile of released PG molecules.

**FIG 3:**
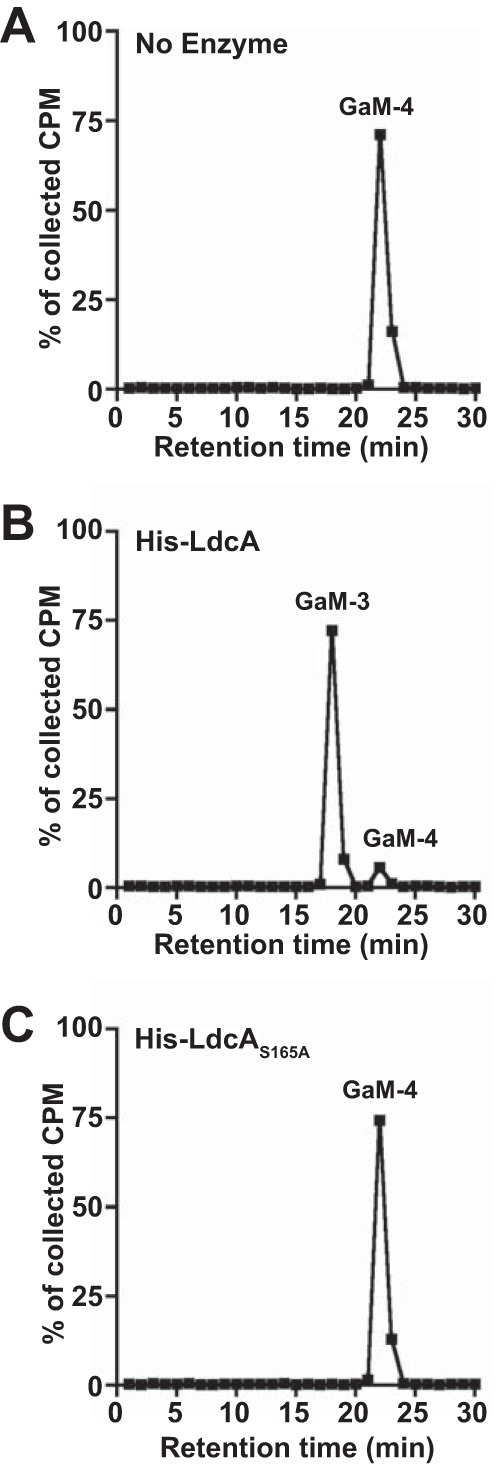
LdcA has l,d-carboxypeptidase activity on PG monomers. (A to C) Radiolabeled tetrapeptide monomer fragments were provided as the substrate in a 2-h reaction with 25 nM (100 ng) N-terminally His-tagged wild-type LdcA (B) or LdcA_S165A_ (C) or in a control reaction with no enzyme (A). Reaction products were separated by reversed-phase HPLC. One-minute fractions were collected and measured by liquid scintillation counting. Counts from each run are displayed as a percentage of the total cpm collected in all HPLC fractions from that run. Enzyme reaction results are representative of at least three independent tests of LdcA enzyme activity on PG monomer. GaM-3, GlcNAc-anhydro-MurNAc-tripeptide monomer; GaM-4, GlcNAc-anhydro-MurNAc-tetrapeptide monomer.

The nature of the PG fragments released by GC strains with mutations in *ldcA* implied that this enzyme has l,d-carboxypeptidase activity. To test this prediction, a 6×His-tagged version of gonococcal LdcA was overexpressed and purified from *E. coli*, along with versions carrying individual alanine substitutions in each of the putative active site residues (S165A, E260A, and H331A) (Fig. S3A). [^3^H]glucosamine-labeled GlcNAc-anhMurNAc-tetrapeptide PG was used as the substrate for 100 ng of purified wild-type LdcA, LdcA_S165A_, LdcA_E260A_, or LdcA_H331A_ in 2-h reactions performed at 37°C. Following reactions performed with LdcA, products were separated by rpHPLC and the fractions were analyzed for radioactivity. PG monomer from a control reaction performed without enzyme eluted, as expected, at 22 min (Fig. 3A), while wild-type LdcA processed nearly all of the tetrapeptide monomer at 22 min to tripeptide monomer that eluted at 18 min (Fig. 3B). Mutation of each of the residues in the putative catalytic triad resulted in loss of activity (Fig. 3C and S3B and C). Increasing the amount of the point mutant enzymes by 10-fold (adding 1 μg/reaction) did not restore wild-type activity (Fig. S3D to F). By all indications, gonococcal LdcA is a serine protease, capable of cleaving anhydrotetrapeptide PG monomers to anhydrotripeptide PG monomers and responsible for the normal production of the tripeptide monomer fragments released by GC.

10.1128/mBio.01464-17.3 FIG S3  Additional point mutant versions of LdcA are inactive. (A) Coomassie-stained SDS-PAGE gel of purified N-terminally His-tagged wild-type and point mutant versions of LdcA used for enzyme activity assays. One microgram of protein was loaded per lane. (B and C) Radiolabeled tetrapeptide monomer fragments were provided as the substrate in 2-h reactions performed with 25 nM (100 ng) N-terminally His-tagged LdcA_E260A_ or LdcA_H331A_. (D to F) Reactions were performed as described above but with 250 nM (1 μg) N-terminally His-tagged LdcA_S165A_, LdcA_E260A_, or LdcA_H331A_. (B to F) Reaction products were separated by reversed-phase HPLC. One-minute fractions were collected and measured by liquid scintillation counting. Counts from each run are displayed as a percentage of the total cpm collected in all HPLC fractions from that run. Download FIG S3, EPS file, 1.2 MB. Copyright © 2017 Lenz et al. 2017 Lenz et al. This content is distributed under the terms of the Creative Commons Attribution 4.0 International license.

### LdcA controls the production of soluble PG dimer.

**FIG 4:**
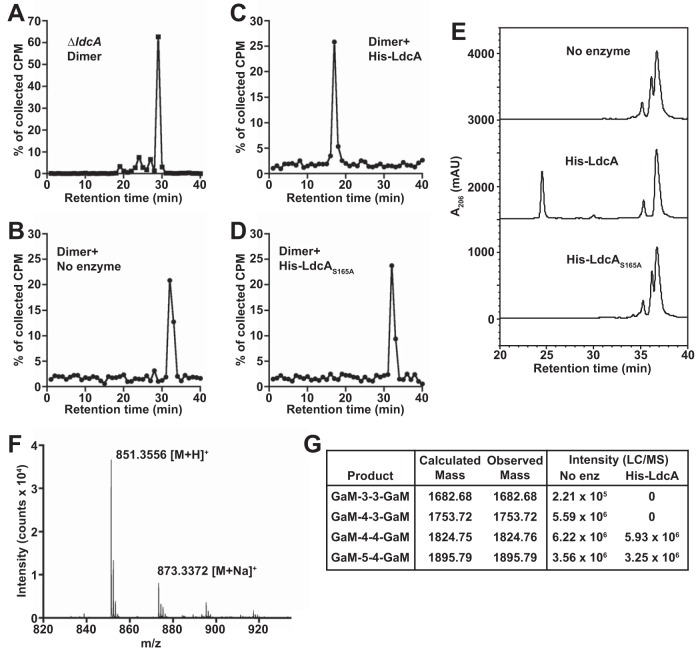
LdcA processes peptide-linked PG dimers to PG monomers. (A) The radiolabeled PG dimer fraction was obtained by size exclusion chromatography analysis of a Δ*ldcA* strain (Fig. 2B), and 20,000 cpm of dimers were separated by reversed-phase HPLC. (B to D) Radiolabeled peptide-linked PG dimer from an *ldcA* mutant was provided as the substrate in 2-h reactions with no enzyme (B) or 25 nM (100 ng) N-terminally His-tagged wild-type LdcA (C) or LdcA_S165A_ (D). Reaction products were separated by reversed-phase HPLC. Fractions were collected and measured; count data are displayed as described for Fig. 3. Enzyme reaction results are representative of at least two independent tests of LdcA enzyme activity on PG dimers. (E) Peptide-linked PG dimers were isolated from an LtgA digestion of *ldcA*_S165A_ *pacA*_H329Q_ sacculi and provided as the substrate in 4-h reactions performed with 25 nM (100 ng) N-terminally His-tagged wild-type LdcA or LdcA_S165A_ or in a control reaction performed with no enzyme. mAU, milliabsorbance units. (F) Time of flight mass spectrometry analysis of the product peak appearing at ~25 min in the His-LdcA reaction whose results are presented in panel E. The *m/z* values for major observed ions are indicated on the graph. (G) Separate digestions of dimer with wild-type LdcA and a no-enzyme (No enz) control (as described for panel E) were performed and analyzed by LC/MS. The observed masses corresponding to peptide-linked dimers present after LdcA reactions are indicated with their intensity values (in arbitrary units). GaM, *N*-acetyl-glucosaminyl-1,6-anhydro-*N*-acetyl-muramyl. Numbers indicate the length of peptide chains attached to each GaM unit. LC/MS analysis was performed two times with similar results; one representative set of intensity values is displayed.

Strains with disruptions in *ldcA* released a much larger proportion of PG multimer fragments than a wild-type strain (Fig. 2A to C), a finding that was entirely unexpected based on the known activity of other characterized l,d-carboxypeptidases. The PG fragments that eluted at ~150 ml in tandem size exclusion chromatography were PG dimers with two general configurations: (i) anhydrotetrasaccharides with two attached peptide chains (glycosidically linked dimers) and (ii) anhydrodisaccharides with cross-linked peptide chains (peptide-linked dimers). Peptide cross-linking is typically observed as a bond between the terminal d-Ala of one chain and the *m*DAP of another, often noted as a 4-to-3 linkage in reference to the position of the linked partners in the peptide chain. In wild-type GC, d-Ala-*m*DAP linkages are the most common cross-links, but *m*DAP-*m*DAP linkages are also observed (27, 32). The possible chain length combinations (tetrapeptide-tetrapeptide, tetrapeptide-tripeptide, tripeptide-tripeptide) for both the glycosidically linked and peptide-linked dimers are the reason that several closely related products were observed in the released dimer fraction from wild-type GC (Fig. S4A). To determine how PG dimer release changes in *ldcA* mutants, dimer was isolated from size exclusion chromatography separation of released PG from the Δ*ldcA* mutant and each of the three *ldcA* active site mutants. When dimer fractions were separated by rpHPLC on a 5% to 25% acetonitrile gradient over 60 min, the *ldcA* mutants were shown to release primarily a single dimer species (Fig. 4A and S4B to D). The major observed dimer species is resistant to mutanolysin treatment (which cleaves the minority glycosidically linked species), indicating that the major released species was likely a peptide-linked dimer (data not shown).

10.1128/mBio.01464-17.4 FIG S4  LdcA releases primarily a single peptide-linked dimer species. (A to D) The radiolabeled PG dimer fraction was obtained by size exclusion chromatography analysis (Fig. 2C and S2A) of (A) wild-type (MS11), (B) *ldcA*_S165A_, (C) *ldcA*_E260A_, and (D) *ldcA*_H331A_ strains. Twenty thousand cpm of dimers were separated by reversed-phase HPLC. One-minute fractions were collected and measured by liquid scintillation counting. Counts from each run are displayed as a percentage of the total cpm collected in all HPLC fractions from that run. Download FIG S4, EPS file, 0.7 MB. Copyright © 2017 Lenz et al. 2017 Lenz et al. This content is distributed under the terms of the Creative Commons Attribution 4.0 International license.

The relatively large proportion of peptide-linked dimer released from *ldcA* mutants compared to the results seen with the WT strain implies that LdcA somehow participates in the clearance of peptide cross-links during fragment release, either directly or by interaction with an endopeptidase whose activity is LdcA dependent. To test whether LdcA can act directly on peptide-linked PG dimer, [^3^H]glucosamine-labeled dimer was used as the substrate for purified LdcA and LdcA_S165A_ (Fig. 4B to D). LdcA was able to cleave the majority dimer species to a product with a retention time identical to that seen with the tripeptide monomer (Fig. 4C), while LdcA_S165A_ had no activity (Fig. 4D). These results suggest that the identified carboxypeptidase active site is also used for endopeptidase activity. To further test if peptide-linked dimers are a substrate for LdcA, we generated unlabeled peptide-linked dimer substrate by digesting nonacetylated sacculi from an *ldcA* mutant (*ldcA*_S165A_ *pacA*_H329Q_) with LtgA, one of the lytic transglycosylases responsible for generating released PG fragments (containing 1,6-anhydro-MurNAc) (32). Peptide-linked dimer fragments were then purified by rpHPLC, and mixed dimer species were provided as the substrate in reactions with LdcA and LdcA_S165A_. When products of digestions were separated by rpHPLC, we observed that LdcA, but not inactive LdcA_S165A_, caused the disappearance of one of the three observed dimer species and that the appearance of a single product was retained where monomer would be expected (Fig. 4E). The single product at ~25 min was confirmed to be tripeptide monomer by mass spectrometry (expected value, 850.34; observed values, 851.35 [M + H]^+^, 852.35 [M + 2H]^2+^, and 873.34 [M + Na]^+^) (Fig. 4F), indicating that LdcA can process some cross-linked dimers to monomers. To determine which dimer species were present prior to digestion and which disappeared upon LdcA treatment, an additional LdcA digestion and a matched no-enzyme control were analyzed by liquid chromatography-mass spectrometry (LC/MS). The reaction mixtures used in reactions performed without enzyme (substrate only) contained primarily GaM-4-3-GaM, GaM-4-4-GaM, and GaM-5-4-GaM (with very low levels of GaM-3-3-GaM). Digestion with LdcA eliminated GaM-3-3-GaM and GaM-4-3-GaM without a major impact on GaM-4-4-GaM and GaM-5-4-GaM. This finding indicates that it is likely that LdcA cannot “trim” peptide stems of cross-linked GaM-4-4-GaM dimers but can access and cleave an *m*DAP-*m*DAP cross-link or an *m*DAP–d-Ala cross-link or both when at least one stem is already tripeptide in length. The activity of LdcA for cleaving peptide-linked dimers to monomers explains why *ldcA* mutants release an unusually large amount of PG dimer fragments.

### Loss of LdcA eliminates uncrosslinked tripeptide stems from the assembled sacculi.

Cell wall assembly utilizes the precursor UDP-MurNAc-pentapeptide, which can be assembled by two different routes: (i) *de novo* peptidoglycan biosynthesis in which amino acids are sequentially added to the sugar moiety and (ii) peptidoglycan recycling in which Mpl adds UDP-MurNAc to existing peptide stems imported to the cytoplasm from the periplasm. For PG recycling to occur, reused stems must be made tripeptide in length so that MurF can ligate a single d-Ala-d-Ala unit to the carboxy-terminal *m*DAP to restore UDP-MurNAc-pentapeptide. In *E. coli*, *ldcA* mutants are unable to utilize PG recycling to generate UDP-MurNAc-pentapeptide, accumulating a dead-end UDP-MurNAc-tetrapeptide (7, 8). While defects in *ldcA* activity are detrimental for *E. coli* survival in the stationary phase, differences in *E. coli* sacculus composition have been noted to be minimal in the exponential phase (7) or to affect primarily the abundance of particular cross-links that rely on pentapeptides (8). In contrast, the muropeptide profile determined for *C. jejuni* lacking the putatively periplasmic l,d-carboxypeptidase Pgp2 indicates a complete loss of tripeptide-stem PG from the sacculus (9). Since GC LdcA is a serine protease similar to LdcA in *E. coli* but can be exported from the cytoplasm like Pgp2, we wanted to address the issue of how *ldcA* mutation affects the composition of the GC sacculus.

**FIG 5:**
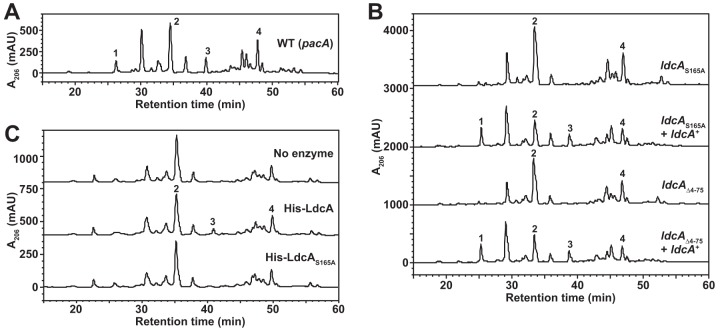
Free tripeptides are lost from GC sacculi without functional LdcA. (A) Mutanolysin-digested sacculi from wild-type strain MS11 with a *pacA*_H329Q_ mutation (nonacetylated PG) separated by reversed-phase HPLC. The indicated peaks represent the following PG monomers: peak 1, GlcNAc-MurNAc-tripeptide; peak 2, GlcNAc-MurNAc-tetrapeptide; peak 3, GlcNAc-anhydro-MurNAc-tripeptide; peak 4, GlcNAc-anhydro-MurNAc-tetrapeptide. (B) Mutanolysin digestions and reversed-phase HPLC of nonacetylated sacculi from *ldcA*_S165A_, *ldcA*_S165A_ + *ldcA*^*+*^, *ldcA*_Δ4−75_, and *ldcA*_Δ4−75_ + *ldcA*^*+*^ strains. (C) Mutanolysin digestions and reversed-phase HPLC of sacculi from strain *ldcA*_S165A_ *pacA*_H329Q_ following an overnight reaction performed with 250 nM (1 μg) N-terminally His-tagged wild-type LdcA or LdcA_S165A_ or a control reaction performed with no enzyme.

To determine if *ldcA* disruption results in differences in PG composition, we introduced the *ldcA*_S165A_ and *ldcA*_Δ4−75_ mutant/complement pairs described above into a GC *pacA*_H329Q_ background (nonacetylated sacculi [33]) and grew these bacteria to the exponential phase. Peptidoglycan sacculi were purified from each strain and digested with mutanolysin, and digested products were separated on rpHPLC. An elution profile from a *N. gonorrhoeae* MS11 *pacA*_H329Q_ strain with wild-type *ldcA* was run for comparison, and the major peaks of interest are indicated as follows: peak 1, reducing tripeptide monomer; peak 2, reducing tetrapeptide monomer; peak 3, anhydrotripeptide monomer; peak 4, anhydrotetrapeptide monomer (Fig. 5A). In PG from both of the *ldcA* mutants, the presence of uncrosslinked reducing and anhydrotripeptide monomer was eliminated (peaks 1 and 3) whereas the proportion of the tetrapeptide monomers increased (peaks 2 and 4), with each of these changes complemented by expression of inducible wild-type *ldcA* (Fig. 5B). To test whether LdcA could act on sacculi to restore either of the uncrosslinked tripeptide monomer species, purified LdcA was added to whole GC sacculi from an *ldcA* mutant (*ldcA*_S165A_, *pacA*_H329Q_), followed by subsequent mutanolysin digestion and rpHPLC as described above. Treatment of sacculi with LdcA results in the appearance of one of the previously lost peaks, representing anhydrotripeptide monomer (peak 3) (Fig. 5C). Mutanolysin digestion of sacculi should produce reducing-end fragments internal to glycan strands and anhydro-end fragments present on the ends of strands. Our data therefore indicate that LdcA can act on assembled sacculi but may be restricted to uncrosslinked peptide stems accessible on the end of strands rather than trimming peptides internal to strands. This conclusion is consistent with our findings from dimer digestion, where LdcA did not appear to trim tetrapeptides that were engaged in cross-links (Fig. 4G). It was also evident from the analysis of dimer digestions that some tripeptide stems exist even in the *ldcA* mutant sacculi, present as peptide-linked 3-to-3 and 4-to-3 dimers. These tripeptide stems are likely the result of l,d-transpeptidases generating cross-links from a donor tetrapeptide stem (34). It is also noteworthy that inactivation of LdcA and signal sequence truncation have similar effects on uncrosslinked tripeptide stems in the sacculus, demonstrating that LdcA must directly act in the periplasm to generate uncrosslinked tripeptide stems and cannot affect the tripeptide stem levels if it is cytoplasm restricted.

### NOD1 and NOD2 activation of NF-κB by soluble *N. gonorrhoeae* products is eliminated without LdcA activity.

**FIG 6:**
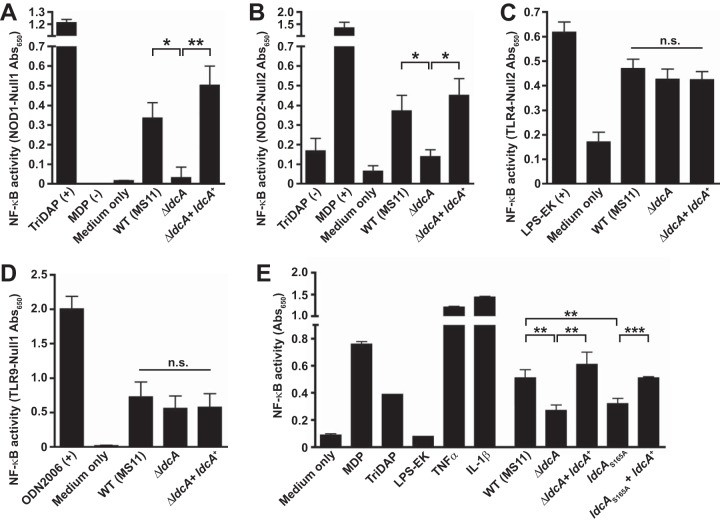
LdcA is responsible for the release of human NOD1 agonist by GC. (A to D) HEK-293 cells overexpressing hNOD1 (A), hNOD2 (B), TLR4 (C), or TLR9 (D) and transfected with an secreted alkaline phosphatase (SEAP) reporter for NF-κB activity (HEK-Blue cells) were exposed to control ligands, cGCBL medium, or cell-free supernatant from growth of wild-type strain MS11, a Δ*ldcA* strain, or a complemented mutant (Δ*ldcA* + *ldcA*^*+*^). SEAP levels were measured colorimetrically at OD_650_, and relative NF-κB activity levels are expressed as the OD_650_ values from HEK-Blue reporter cells, with background OD_650_ values subtracted from those determined for a matched cell line with a SEAP reporter but no construct for NOD/TLR receptor expression (HEK-Blue Null1/2 cells). Reporter cells and Null cells were grown, treated, and measured in parallel for each experiment. Values represent means ± standard errors of averages of results from three independent experiments. (E) HCT116 cells transfected with a secreted alkaline phosphatase (SEAP) reporter for NF-κB activity (HCT116-Dual cells) were exposed to control ligands or cGCBL medium or were infected with ~1 × 10^6^ CFU/ml of pilus^+^ Opa^−^ versions of the following strains: the wild-type strain (MS11), mutant Δ*ldcA*, mutant Δ*ldcA* + *ldcA*^*+*^, mutant *ldcA*_S165A_, and mutant *ldcA*_S165A_ + *ldcA*^*+*^. Measurement of SEAP activity at OD_650_ provides a measurement of relative NF-κB activity levels. Values displayed are the means ± standard deviations of results from triplicate wells determined in one experiment representative of three independent experiments. (A to E) Significance was determined for the bracketed comparisons by unpaired *t* test. *, *P* < 0.05; **, *P* < 0.01; ***, *P* < 0.001; n.s., not significant. TriDAP is a NOD1 agonist (l-Ala-γ-d-Glu-*m*DAP), MDP is a NOD2 agonist (muramyl dipeptide), LPS-EK is a TLR4 agonist (lipopolysaccharide from *E. coli* K-12), and ODN2006 is a TLR9 agonist (CpG oligonucleotide). IL-1β, interleukin-1β; TNF-α, tumor necrosis factor alpha.

PG fragments from bacteria are recognized by eukaryotic intracellular pattern recognition receptors NOD1 and NOD2, which activate downstream inflammatory responses via NF-κB and AP-1 (16, 17). Monomeric PG fragments have been shown to promote ciliated cell death and sloughing in human Fallopian tube explants during infection with GC (23), and the presence of cell-free supernatant alone is sufficient to activate a NOD1-dependent NF-κB response (35). Since human NOD1 (hNOD1) is specific for tripeptide (l-Ala-d-Glu-*m*DAP), whereas the murine NOD1 senses the related tetrapeptide (l-Ala-d-Glu–*m*DAP–d-Ala), we hypothesized that the observed LdcA-dependent changes in PG fragment release would alter the ability of GC to activate NF-κB via hNOD1. To test this hypothesis, we employed HEK-293 cells overexpressing the hNOD1 receptor and carrying an NF-κB reporter (HEK-Blue cells). Cells were treated with conditioned media (normalized to cellular growth) from either WT strain MS11 or each of the *ldcA* mutant-complement pairs described previously. Purified NOD1 agonist TriDAP (l-Ala-d-Glu-*m*DAP) was used as a positive control (25), while negative controls included complete untreated gonococcus base liquid (cGCBL) medium and muramyl dipeptide (MDP; a NOD2 agonist). In all cases, loss of *ldcA* activity resulted in significant loss of hNOD1-dependent NF-κB signaling (Fig. 6A). While NOD2 is activated to a lesser degree than NOD1 by GC supernatant (which is known not to contain the agonist muramyl dipeptide) (35), we also observed an *ldcA*-dependent decrease in NOD2 activation (Fig. 6B). Since GC undergoes spontaneous lysis in culture and GC sacculi can activate NOD2 responses (35), it is possible that without functional LdcA, PG stems are less efficiently broken down from tripeptide stems to the dipeptide stems that form the basis of recognition by NOD2. To ensure that loss of LdcA function was not having broader effects on the production of immune agonists by GC, parallel experiments were performed on a cell line with a Toll-like receptor 4 (TLR4) reporter (Fig. 6C) and a cell line with a TLR9 reporter (Fig. 6D). There appear to have been no significant LdcA-dependent changes in the lipooligosaccharide (LOS) sensed via TLR4 or in DNA secretion/cell lysis-dependent DNA release sensed through TLR9.

Since the HEK-Blue system relies on overexpression of specific receptors, we also employed a similar secreted embryonic alkaline phosphatase (SEAP) reporter cell line (HCT116-Dual) that has only physiological levels of NOD1 and NOD2 (and does not express TLR2 and TLR4) (36). HCT116 cells are also colorectal epithelial cells, chosen for their physiological relevance to a common site of GC infection (37). Interestingly, these cells do not respond to addition of GC-conditioned media, as HEK-Blue cells do, but do respond to infection with GC (data not shown). Infections of HCT116-Dual cells with wild-type GC or complemented *ldcA* mutants resulted in more NF-κB reporter activation than was seen with infections with strains containing either an in-frame *ldcA* deletion or the *ldcA*_S165A_ active site point mutation. Using each of the reporter systems described above, we concluded that *ldcA* is responsible for the production of hNOD1 agonist PG fragments and that eliminating this activity attenuates the host inflammatory response to GC.

## DISCUSSION

The ability of *N. gonorrhoeae* to release tripeptide-stem PG fragments from the breakdown of a tetrapeptide-rich sacculus has been a curious footnote for almost 40 years (24, 27). The original hypotheses for why tripeptide fragments are enriched included the idea that GC hexosaminidase preferred removing tripeptide stems from sacculi or that an unidentified d,l-carboxypeptidase was somehow released from cells to act on fragments in the medium. It would be another 20 years before an enzyme with l,d-carboxypeptidase activity was characterized, though it was a cytoplasmic enzyme critical for the recycling of PG fragments and the survival of *E. coli* in the stationary phase (8). Nearly 20 years on from that discovery, crystal structures of l,d-carboxypeptidases from Gram-negative alphaproteobacteria (6), gammaproteobacteria (5), epsilonproteobacteria (11), and Gram-positive bacteria (15) have been completed. These studies revealed a range of catalytic options for cleaving the l-d bond between *m*DAP and d-Ala, with some organisms relying on serine protease activity, others on a repurposed l,d-transpeptidase, and yet others on LAS-family metallopeptidases. The Gram-negative betaproteobacteria did not yet have a characterized l,d-carboxypeptidase—there was only a prediction that *N. meningitidis* had an enzyme similar in structure to the serine protease l,d-carboxypeptidase of *P. aeruginosa* (38). In this work, we define NGO1274 in *N. gonorrhoeae* as an l,d-carboxypeptidase and propose naming this gene *ldcA*. *N. gonorrhoeae* LdcA shares serine protease active site residues with the homologous serine protease l,d-carboxypepdidases from alphaproteobacteria and gammaproteobacteria, and we demonstrate that mutating those same residues in GC LdcA disrupts PG fragment release and NOD activation.

A critical difference between GC LdcA and all other serine protease l,d-carboxypeptidases is the addition of a TAT-dependent signal sequence that facilitates export of GC LdcA to the periplasmic space. LdcA contains a predicted signal peptidase II cleavage site and the “lipobox” sequence Leu-Gln-Ala-Cys (39), suggesting that LdcA is an outer membrane lipoprotein similar to other gonococcal PG-degrading enzymes such as LtgA and LtgD (32). It should be noted, however, that the observed soluble form of LdcA could be present in the cytoplasm and/or the periplasm. Periplasmic localization is predicted for the nonhomologous l,d-carboxypeptidases from the epsilonproteobacteria (Pgp2 and Csd6) (9, 11), although these sequences appear to contain Sec-dependent rather than TAT-dependent signal peptides, implying that functional GC LdcA is able to fold in the cytoplasm prior to export. Folding in the cytoplasm could make some LdcA available for cytoplasmic PG recycling (similarly to cytoplasm-restricted LdcA homologs). The presence of an extended N-terminal leader and TAT-dependent signal sequence on l,d-carboxypeptidases is not a common feature of all betaproteobacteria. LdcA homologs within the human-pathogenic *Burkholderiaceae* have N termini comparable to those seen with the alphaproteobacteria and gammaproteobacteria, while the *Neisseriaceae* (including the genera *Neisseria*, *Kingella*, and *Eikenella*) have extended N-terminal regions. It is not known at this time if other genera within the *Neisseriaceae* share any other aspects of PG metabolism (including soluble PG fragment release) with *Neisseria* or if any other aspect of *Neisseriaceae* biology necessitates periplasmic localization of LdcA.

When purified LdcA from *E. coli* was first tested on a variety of PG substrates, it had the greatest activity on free tetrapeptide stems (without sugars), less activity on disaccharide-tetrapeptide/anhydrodisaccharide-tetrapeptide (PG monomer) fragments, and no detectable activity on assembled murein sacculi or on *bis*-disaccharide-tetrapeptide/*bis*-anhydrodisaccharide-tetrapeptide (peptide-linked PG dimers) (8). This result is perhaps not surprising as cytoplasm-restricted l,d-carboxypeptidases would presumably never encounter assembled sacculi or peptide-linked dimers as substrates. While we do not yet know exactly how GC LdcA differs structurally from other LdcA homologs, it appears able to process some, but not all, peptide-linked dimer substrates (with a preference for 4-to-3 cross-links over 4-to-4 or 4-to-5 cross-links). Furthermore, our data provide several insights into when and where LdcA is able to act. First, LdcA substrate must either be soluble (already liberated by lytic transglycosylases) or accessible on the end of a strand, as evidenced by its limited activity on assembled sacculi (and the presence of tetrapeptide stems as a majority of the wild-type sacculus). Second, GC LdcA does not appear to trim the tetrapeptide stems of 4-to-4 cross-linked fragments (an activity performed by Pgp2, a structurally unrelated l,d-carboxypeptidase) (9). Instead, the dimer substrate must have at least one stem that is already tripeptide in length. Some of the peptide-linked dimers in the wild-type sacculi have tripeptide stems, formed by transpeptidases during sacculus assembly (40). Aside from the unique ability to cut peptide-linked dimers, GC LdcA shares most other aspects of substrate profile with the alphaproteobacterial and gammaproteobacterial serine protease LdcA homologs. Characterization of *Novosphingobium aromaticivorans* LdcA included a side-by-side comparison with *E. coli* LdcA (6), which demonstrated similar patterns of substrate recognition by *N. aromaticivorans* and *E. coli* on tetrapeptide, MurNAc-tetrapeptide, UDP-MurNAc-tetrapeptide, and anh-disaccharide-tetrapeptide (closely replicating the *E. coli* results from Templin et al.) (8). Das et al. then extended the analysis to confirm that both tested versions of LdcA have little to no activity on UDP-MurNAc-pentapeptide or a tetrapeptide stem with l-lysine in the third position (instead of *m*DAP) (6). LdcA from GC is similarly incapable of cleaving UDP-MurNAc-pentapeptide or a synthetic tetrapeptide with l-Lys in the third position (data not shown).

Due to the critical nature of PG for most bacteria and the evolutionary conservation of its general structure, higher eukaryotes have evolved innate immune receptors to detect and respond to the presence of PG. The intracellular pathogen *Shigella flexneri* was the first shown to activate a cytosolic receptor known as CARD4 (Nod1) (41), which detects PG fragments generated by both intracellular and extracellular bacterial infections (42). In humans, NOD1 detects PG fragments of various sizes containing the *m*DAP residue (25), while NOD2 detects muramyl dipeptide (MDP) (17) as well as PG monomer fragments with reducing-end MurNAc generated by host lysozyme (rather than the 1,6-anhydro-MurNAc generated by bacterial lytic transglycosylases) (43). The release of PG fragments would therefore seem to present a liability for bacteria during encounters with the host immune system, where detection by NOD1 and NOD2 initiates inflammatory responses via the transcription factors NF-κB and AP-1. By efficiently recycling PG fragments during growth and importing PG fragments to the cytoplasm, Gram-negative bacteria can avoid generating high levels of NOD1 agonist in the periplasm that risk being lost from the cell and detected by the host. Gonococci, rather than limiting the loss of NOD1 agonist, shed NOD1 agonist PG during normal growth in a manner that is dependent on not only LdcA activity but specifically periplasmic LdcA activity. LdcA itself is not essential in GC, and most other bacteria seem to have evolved to keep LdcA in the cytoplasm. It follows, therefore, that the release of NOD1 agonist must confer an adaptive advantage to GC or must at least have an effect that is overcome by another adaptation. For example, inflammation itself draws neutrophils to the site of infection, an encounter in response to which GC has adapted several mechanisms to survive (44). The result of the struggle between invading GC and the host innate response includes generation of a purulent exudate containing bacteria. The presence of live bacteria present in exudate and of bacteria surviving inside neutrophils increases the probability of transmission (45), possibly making inflammation beneficial for GC. The functions of NOD1 activation are also not limited to the induction of proinflammatory responses. NOD1 activation can promote autophagy and modulate apoptosis (46–48), giving GC the potential to manipulate host cell death pathways to avoid immune clearance. Further study of the role of soluble NOD agonist release, facilitated by LdcA, at the level of individual host cell responses is necessary to gain a fuller understanding of how GC maintains success as a pathogen while antagonizing the human immune system.

## MATERIALS AND METHODS

### Bacterial strains and growth.

*N. gonorrhoeae* strain MS11 was used as our laboratory wild-type strain, and all mutants used in this study are derived from this strain (49). All *N. gonorrhoeae* strains were grown under conditions of 37°C and 5% CO_2_ on gonococcal base (GCB) agar plates (Difco) containing Kellogg’s supplements (50, 51). Liquid cultures were started from overnight plates and grown in gonococcal base liquid medium (GCBL) containing Kellogg’s supplements and 0.042% NaHCO_3_ (complete GCBL [cGCBL]) for no more than 3 h, with agitation, as described previously (52). Chloramphenicol (cam) was used at 5 to 10 μg/ml in GCB plates for selection of the insertion of inducible constructs at the *aspC*-*lctP* chromosomal site. Erythromycin (erm) was used at 10 μg/ml (Erm_10_) in GCB plates for selection of the insertion of inducible constructs at the *iga-trpB* chromosomal site (30). Gene expression was induced in gonococci from the *aspC*-*lctP* chromosomal site with 1 mM isopropyl-β-d-thiogalactopyranoside (IPTG) during growth in culture (53), and 0.1 mM IPTG was added to tissue culture media during infection experiments to maintain expression. Gene expression was induced in gonococci from the *iga-trpB* chromosomal site with 40 ng/ml anhydrotetracycline (aTc). *E. coli* strains were grown on Luria agar plates or in Luria broth (LB) at 37°C. Antibiotics were used at the following concentrations for *E. coli* experiments: ampicillin (amp) at 50 to 100 μg/ml; kanamycin (kan) at 40 μg/ml; erythromycin (erm) at 500 μg/ml; and chloramphenicol (cam) at 25 μg/ml. RapidTrans chemically competent TAM1 *E. coli* (Active Motif, Inc.) was used for all cloning, and BL21 Star (DE3) *E. coli* (Invitrogen) was used for protein purification.

### Plasmid and strain construction.

To generate constructs for protein purification, the *ldcA* gene lacking its predicted signal sequence was amplified from *N. gonorrhoeae* MS11 chromosomal DNA using primers ldcANheFWD (5′ CGAGCTAGCGGCACATCCGCCACATCCGTTC 3′) and ldcAXhoREV (5′ AGTCTCGAGGCGGGTTTATTCCGAAATATC 3′), which introduce NheI and XhoI sites, respectively (underlined). This insertion was digested with NheI and XhoI and ligated into similarly digested pTEV5 (54) to generate pJDL1, which adds an in-frame N-terminal 6×His tag to *ldcA*. To generate point mutant versions of *ldcA* for cloning into pTEV5, individual point mutations were introduced on overlapping outward-facing primers from each desired mutation site. Flanks 5′ and 3′ of the mutation site were then amplified with pairs consisting of an outward facing primer and one of the primers noted above. For the S165A mutation, the 5′ flank was amplified with ldcANheFWD and JLDNA25 (5′ ACGTC**GGC**GAATCCGAAAAACAG 3′) and the 3′ flank was amplified with JLDNA26 (5′ GATTC**GCC**GACGTATGCGCCGTC 3′) and ldcAXhoREV. For the E260A mutation, the 5′ flank was amplified with ldcANheFWD and JLDNA28 (5′ GACATC**TGC**GAGGAACAAAATGC 3′) and the 3′ flank was amplified with ldcAXhoREV and JLDNA30 (5′ TTCCTC**GCA**GATGTCGGCGAACA 3′). For the H331A mutation, the 5′ flank was amplified with ldcANheFWD and JLDNA31 (5′ GGCAATG**GCG**CCGAACGGGAAGC 3′) and the 3′ flank was amplified with ldcAXhoREV and JLDNA32 (5′ TTCGG**CGC**CATTGCCGACAAAAT 3′). In each of the primers described above, the bases indicated in bold represent the codon changed to alanine. For each of the changes described above, the paired 5′ and 3′ flanks were used as the template with ldcANheFWD and ldcAXhoREV primers in an overlap extension PCR and the resulting product was cloned into pTEV5 as noted above to generate pJDL21 (pTEV5::*ldcA*_S165A_), pJDL24 (pTEV5::*ldcA*_E260A_), and pJDL25 (pTEV5::*ldcA*_H331A_). All of the plasmids described above were transformed into *E. coli* and screened by colony PCR for the correct insertion size, and the insertions were sequenced to confirm the desired assembly.

Constructs used to introduce point mutations into *ldcA* in the gonococcal chromosome were assembled using a strategy similar to that described above to generate the insertions for the shuttle vector pIDN3 (55). To introduce an S165A mutation, the 5′ flank was amplified with ldcA-Pst-F (5′ TGACTGCAGAAATGACCGCCCAACCCTGC) and JLDNA25 and the 3′ flank was amplified with JLDNA27 (5′ GATTC**GCC**GACGTATGCGCCGTCCAGCTGGC 3′) and ldcA-SalI-R (5′ AATGTCGACTGAACACTTCCGCCGTTCAC). The JLDNA27 primer introduces a silent mutation (underlined) that generates a PvuII site for screening. For the E260A mutation, the 5′ flank was amplified with ldcA-Pst-F and JLDNA29 (5′ ACATC**TGC**GAGGAACAAAATGCCGCCGTCGATATCGGG 3′) and the 3′ flank was amplified with ldcA-SalI-R and JLDNA30. The JLDNA29 primer introduces a silent mutation (underlined) that generates an EcoRV site for screening. For the H331A mutation, the 5′ flank was amplified with ldcA-Pst-F and JLDNA31 and the 3′ flank was amplified with ldcA-SalI-R and JLDNA32. The JLDNA31 and JLDNA32 primers introduce a silent mutation (underlined) that generates a HaeII site for screening. As described above, paired 5′ and 3′ flanks were used as the template with ldcA-Pst-F and ldcA-SalI-R primers in an overlap extension PCR, and the resulting products were ligated into pIDN3 following digestion of the vector and the insertions with PstI and SalI. The resulting constructs were pJDL23 (pIDN3::*ldcA*_S165A_), pJDL30 (pIDN3::*ldcA*_E260A_), and pJDL31 (pIDN3::*ldcA*_H331A_). All of the plasmids described above were transformed into *E. coli*, screened, and confirmed by sequencing as described above. To generate mutations on the chromosome of *N. gonorrhoeae*, 2 μg of each plasmid was linearized with PciI in an overnight digestion and was introduced into piliated *N. gonorrhoeae* MS11 by the spot transformation method (52). To select for double-crossover replacement of WT *ldcA* with each of the desired point mutations (rather than insertion-duplication of pIDN3), transformed strains were streaked for isolation with no antibiotic selection. Individual colonies were screened by first PCR amplifying *ldcA* using ldcA-Pst-F and ldcA-SalI-R and then digesting a portion of each PCR product with the appropriate enzyme as noted above. The PCR products that showed digestion, indicating acquisition of a silent restriction site, were sequenced to confirm acquisition of the desired mutation in the gonococcal chromosome. The resulting strains were designated JL507 (*ldcA*_S165A_), JL510 (*ldcA*_E260A_), and JL511 (*ldcA*_H331_).

To generate an in-frame deletion of *ldcA* from the gonococcal chromosome, a construct was first made containing the 500 bp upstream from the *ldcA* coding sequence, which includes the start codon, and 500 bp downstream, which includes the stop codon, joined by a KpnI restriction site. To assemble this plasmid, the upstream flanking region was amplified with ldcA-Pst-F and JLDNA58 (5′ GGGGTACCGGTCATAATTTCCCTTTCATACGG 3′) and the downstream region was amplified with JLDNA59 (5′ GGGGTACCTAAACCCGCAAACGGACAA 3′) and ldcA-Sal-R (KpnI site underlined). Flanking region products were sequentially digested with KpnI followed by PstI or SalI as appropriate, and pIDN3 was digested with PstI and SalI simultaneously. The insertions and vector were assembled in a three-way ligation, transformed into *E. coli* as described above, and selected on LB_Erm_. The screened and sequence-confirmed plasmid was designated pJDL48 and spot transformed into *N. gonorrhoeae* as indicated above. Colonies were screened by PCR for the loss of *ldcA*, as indicated by an ~1-kb PCR product generated from primers outside those used for cloning (JLDNA72 [5′ CTATCAGAAATTGCCCTACCG 3′] and JLDNA73 [5′ CCCTGTTCTATATCCAAGGTTTG 3′]). PCR products were secondarily screened for KpnI sensitivity, and those clones were sequenced to confirm *ldcA* deletion in the gonococcal chromosome. The resulting Δ*ldcA* strain was designated JL539.

To generate a truncated version of *ldcA* lacking its putative signal sequence but containing native sequence upstream of the start, *ldcA* was amplified from GC strain MS11 with primers ldcA-Pst-F and ldcA-Sal-R (described above), digested with PstI and SalI, and ligated into similarly digested pIDN1 (55). The resulting construct was then used in a PCR with primers ldcAsigseqBsaF (5′ TTTGGTCTCTATGGGCACATCCGCCACATCCGTTC 3′) and ldcAsigseqBsaR (5′ CTTGGTCTCCCCATAATTTCCCTTTCATACGGTTTC 3′). This reaction, which proceeded around the plasmid but omitted bases 4 to 75 of *ldcA*, generated a fragment that was digested with BsaI and ligated to generate pIDN1::*ldcA*_Δ4−75_, designated pKH162. The pKH162 plasmid was linearized and introduced into MS11 by spot transformation (as described above). The resulting colonies were screened by PCR for truncations of *ldcA* at its native site and confirmed by sequencing. The resulting strain was designated KH619. To express wild-type *ldcA* in all *ldcA* disruption backgrounds, an IPTG-inducible complementation construct was made by amplifying *ldcA* from MS11 with the primers ldcA-XbaF (5′ TGTCTAGAGCCGATGCCGTCTGAAACCG 3′) and ldcA-XhoR (5′ CGTCTCGAGTGCAATCTGCGTTGCCATCC 3′). The resulting PCR product was digested with XbaI and XhoI and then ligated into identically digested pKH37 (56). The resulting plasmid, pKH151, was linearized with SapI and then spot transformed (as described above) into (i) KH619 (*ldcA*_Δ4−75_) to form strain KH620, (ii) JL507 (*ldcA*_S165A_) to form strain JL508, and (iii) JL539 (Δ*ldcA*) to form strain JL540.

For use in subcellular localization experiments, constructs were generated with C-terminal hemagglutinin (HA)-tagged versions of full-length *ldcA* and *ldcA* without its predicted signal sequence. Primer JLDNA64 (5′ CGAGCTCTGAAAGGGAAATTATGACCGAACCG 3′) contains 13 bases upstream of *ldcA*, including a putative Shine-Dalgarno sequence and a SacI site (underlined), while JLDNA9 (5′ GACTAGTTTA**AGCGTAATCTGGAACATCGTATGGGTA**TCCACCTTCCGAAATATCGGCAACAC 3′) adds a C-terminal two-glycine spacer (italic), HA tag (bold), and SpeI site (underlined) to the end of *ldcA*. Primer JLDNA65 (5′ CGAGCTCTGAAAGGGAAATTATGGGCACATCCGCCACATC 3′) also contains a SacI site (underlined) and the same upstream region native to *ldcA* with the start codon but omits the sequence between the start and the putative signal peptidase cleavage site (bases 4 to 75). JLDNA64 and JLDNA65 were used with JLDNA9 to amplify the full-length and truncated *ldcA* insertions, respectively. The insertions were digested with SacI and SpeI and ligated into similarly digested pMR68, a vector with an aTc-inducible promoter capable of integration into the *N. gonorrhoeae* chromosome at the *iga-trpB* complementation site (30). Completed plasmids were transformed into *E. coli*, selected on LB_kan_, PCR screened for correctly sized insertions, and confirmed by sequencing. Plasmid pMR68::RBS-*ldcA*-HA (wild type) was designated pJDL53, and pMR68::RBS-*ldcA*_Δ4−75_-HA (truncated) was designated pJDL54. Plasmids were introduced into the *iga-trpB* site in strain JL551 by spot transformation as noted above. JL551 was generated by introduction of plasmid pMR58 (containing an IPTG-inducible *traW*-*phoA* fusion, a chloramphenicol acetyltransferase gene [*cat*], and homology to the *lctP-aspC* complementation site) in the JL539 (Δ*ldcA*) background. CAT was used as a cytoplasmic control in subcellular fractionation.

### Subcellular fractionation and Western blotting.

Fractionation of *N. gonorrhoeae* was performed essentially as described previously, using previously published controls (PilQ, SecY, and CAT) for separation of the outer membrane, inner membrane, and soluble fractions, respectively (57). Briefly, for outer membrane preparation, strains JL556 (Δ*ldcA*, aTc-inducible *ldcA* at the *iga-trpB* site, CAT gene at the *lctP*-*aspC* site) and JL557 (Δ*ldcA*, aTc-inducible truncated *ldcA* at the *iga-trpB* site, CAT gene at the *lctP*-*aspC* site) were grown on GCB plates overnight prior to passage on 16 GCB plates/strain containing Erm_10_, 1 mM IPTG, and 40 ng/ml aTc. Following overnight incubation, cells were swabbed into phosphate-buffered saline (PBS), pelleted, and suspended in cold 100 mM lithium acetate–200 mM lithium chloride–10 mM EDTA at pH 6.0 (LiAc/LiCl buffer). Samples were rocked for 10 min at room temperature and outer membrane vesicles prepared by repeated aspiration through a needle. Cells were removed by centrifugation, supernatants were harvested and filtered, and then filtrates were subjected to ultracentrifugation at 150,000 × *g* in a Beckman TLA-110 rotor for 2 h at 4°C. In the only significant modification of the published methods, the outer membrane pellet was washed once with 3 ml cold LiAc/LiCl buffer and ultracentrifuged again as described above to recover the final outer membrane fraction.

For total membrane and soluble fraction preparation, 4 3-ml cultures in cGCBL medium under the conditions of selection and induction described above were grown from a single overnight GCB plate of each strain. Bacteria were harvested, washed, sonicated, pelleted, and filtered as described previously (57). Cleared lysate was then subjected to ultracentrifugation as described above. Following ultracentrifugation, 800 μl of each soluble fraction was removed and placed in a new ultracentrifuge tube, while the pellet (total membrane fraction) was washed with an equivalent amount of 0.01 M Tris-HCl (pH 7.0). Soluble and total membrane fractions were then subjected to a second ultracentrifugation, as described above. Upon completion of ultracentrifugation, the supernatant was discarded from the total membrane fraction, and pellets were suspended in 50 μl of water. A 500-μl volume was removed from the soluble fractions and processed with a centrifugal filter with a 10-molecular-weight cutoff (MWCO) and concentrated for 15 min at 10,000 × *g*.

The protein content of all fractions was determined by Bradford assay, and samples were normalized with water to 4 μg of protein in a 20-μl volume prior to addition of 4 μl of Lammeli buffer and denaturation by boiling (~5 min). Samples were loaded on two 12% SDS-polyacrylamide gels, one with 500 ng (3 μl) and one with 3 μg (21 μl), to account for differences in the levels of detectability of control targets and HA, respectively. Samples were separated by SDS-PAGE and transferred to polyvinylidene difluoride (PVDF) membranes (BioRad), where they were blocked with 5% (wt/vol) milk–Tris-buffered saline containing 0.05% Tween 20 (TBST) for 1 h. Western blotting for HA-tagged proteins was performed as described previously (58). Western blotting for fractionation controls was performed with a previously described control mix (57). All blots were incubated with primary antibodies in 5% milk–TBST for 1 h, followed by three 10-min washes with TBST. Secondary antibodies were diluted 1:25,000 (goat anti-rabbit horseradish peroxidase [HRP] for the control mix) or 1:50,000 (goat anti-mouse HRP for HA) in TBST and incubated for 1 h followed by three 5-min washes. Blots were developed using Clarity ECL Western substrate (Bio-Rad) prior to exposure to film.

### Protein purification.

For purification of the 6× His-tagged LdcA and point mutant derivatives, single colonies of each strain were grown in 20-ml to 30-ml cultures of LB_amp_ broth overnight at 37°C, and each culture was used the following day to seed two 1-liter cultures/strain. Cultures were grown at 37°C until an optical density at 600 nm (OD_600_) of ~0.6 to ~0.8 was reached, at which point 0.3 mM IPTG was added to induce protein production and growth was continued for ~2 h at 37°C. At the conclusion of growth, cells were centrifuged (8,000 × *g*, 10 min, 4°C), washed once with cold PBS, and centrifuged again as described above prior to storage at −80°C. For protein purification, pellets were suspended in 20-ml purification buffer (20 mM Tris-HCl [pH 8], 300 mM NaCl, 20 mM imidazole) and disrupted by three passes through a cold French pressure cell (1,000 lb/in^2^). Unbroken cells were removed by centrifugation (20,000 × *g*, 30 min, 4°C), and cleared lysate was batch bound to 500 μl of preequilibrated His-Select nickel affinity gel (Sigma)–20 mM imidazole for 90 min on ice with gentle agitation. Lysate and resin were loaded on a column, and the flowthrough was collected, followed by four washes (5 ml per wash) with purification buffer–20 mM imidazole. Protein was eluted in 1-ml fractions by stepwise additions of purification buffer containing increasing amounts of imidazole (40, 60, 90, and 120 mM). Samples of flowthrough, wash, and elution fractions were evaluated on a Coomassie-stained 12% SDS-polyacrylamide gel to determine purity and yield. Clean elution fractions were pooled and dialyzed in 500 ml of storage buffer (50 mM Tris-HCl [pH 7.5], 300 mM NaCl, 10% [vol/vol] glycerol) overnight at 4°C with constant stirring and then added to 500-ml fresh buffer for an additional 6 h to remove all imidazole. Protein was stored in aliquots mixed with glycerol to reach a final concentration of 50% (vol/vol) for storage at −20°C.

### Peptidoglycan isolation and sacculus analysis.

For analysis of PG sacculi, an *N. gonorrhoeae* MS11 derivative with a point mutation in the gene encoding PG acetylase *pacA* (*pacA*_H329Q_, designated KH530) was used as the parent strain (33) and *ldcA* mutations were reproduced in this background. For PG isolation, nonpiliated (p-) gonococci were grown from an optical density (OD_540_) of 0.25 for 2.5 h at 37°C in 10× 3-ml liquid cultures of cGCBL. Cultures were then combined and split into 2× 85-ml volumes of prewarmed cGCBL for an additional 2 h of growth, followed by dilution into 800 ml prewarmed cGCBL. The resulting 1-liter cultures were grown to an OD_540_ of 0.8. PG was then purified using the boiling SDS method, essentially as described previously (33, 59). Bacteria were harvested by centrifugation (8,000 × *g*, 10 min, 4°C), washed with phosphate buffer at pH 6.0 (PB), suspended in 10 ml PB, and added dropwise to 10 ml boiling 8% (wt/vol) SDS in an Oak Ridge tube. Samples were boiled for 1 h, cooled, centrifuged (45,000 × *g*, 30 min, 15°C), and subjected to another 1 h of SDS boiling. Samples were cooled and centrifuged as before, and pellets washed a minimum of four times with 10 to 20 ml PB to remove SDS. Following the final wash and centrifugation, pellets were suspended in 500 μl PB, transferred to a 1.5-ml tube with 100 μg pronase, and incubated overnight at 37°C with constant agitation. The following day, PG was again added to 10 ml boiling 8% SDS and boiled for 2 h, followed by a minimum of four washes, as described above. For analysis of sacculi by HPLC, 75 μl from each PG preparation was digested in PB with 0.1 mg/ml mutanolysin at 37°C for 16 to 18 h. Samples were boiled for 10 min to stop digestions and centrifuged at 13,000 × *g* for 10 min to pellet insoluble products. The remaining soluble fraction from each sample was applied to a 10-MWCO column and centrifuged for 15 min at 10,000 × *g* to remove residual protein and undigested PG. Reaction mixtures were separated by reversed-phase HPLC on a Prevail C_18_ column (Grace Vydac) (5 μm pore size, 250 by 4.6 mm) operating at room temperature, at a flow rate of 1 ml/min, and using a linear gradient of 0 to 60% eluent (25% acetonitrile [ACN]–0.05% trifluoroacetic acid [TFA]) in a mobile phase of 0.05% TFA–water. Elution of PG products was monitored by absorption at 206 nm. Identities of peaks of interest were confirmed by matrix-assisted laser desorption ionization[en]time of flight (MALDI-TOF) analysis performed in positive ion mode at the University of Wisconsin—Madison Biotechnology Center Mass Spectrometry/Proteomics Facility as described previously (32).

### Peptidoglycan radiolabeling and characterization of released fragments.

Gonococcal peptidoglycan was metabolically labeled (pulsed) with [6-^3^H]glucosamine, and released fragments were quantitatively assessed following a chase period, essentially as described previously (60). At the conclusion of the pulse-labeling step, bacteria were suspended in 6 ml cGCBL medium and the amount of label incorporated in each culture was measured by liquid scintillation counting of 60 μl of culture. Based on these counts, culture volumes were adjusted to provide equal amounts of labeled cells and growth was continued for 2.5 h at 37°C. At the conclusion of the chase period, cells were pelleted by centrifugation (3,000 × *g*, 5 min), and supernatant containing soluble PG fragments was passed through a 0.22-μm-pore-size filter prior to storage at −20°C. Supernatants were separated on tandem size exclusion chromatography columns using 100 mM LiCl as the mobile phase and collected as 3-ml fractions. Five hundred microliters of each fraction was measured by liquid scintillation counting to determine the presence of radioactive PG fragments. The remaining volumes in fractions containing peaks of interest were pooled and evaporated under vacuum and then desalted by passage through a size exclusion chromatography column with water as the mobile phase. Desalted fractions were checked for radioactivity using liquid scintillation counting, pooled, and evaporated under vacuum prior to suspension in water.

To determine the identity of radiolabeled PG fragments, released fragments collected from the size exclusion chromatography step were separated by reversed-phase HPLC using the C_18_ column and the flow rate parameters detailed above. For fragments corresponding in size to PG monomers, 1 × 10^4^ cpm were loaded in a 100-μl volume and eluted on a linear gradient of 16% to 52% eluent (25% ACN–0.05% TFA) in a mobile phase of 0.05% TFA over 30 min (yielding a 4% to 13% ACN gradient). For fragments corresponding in size to PG dimers, a volume corresponding to 2 × 10^4^ cpm was loaded in a 100-μl volume and eluted on a linear gradient of 25% to 100% eluent (25% ACN–0.05% TFA) in a mobile phase of 0.05% TFA over 60 min (yielding a 5% to 25% ACN gradient). For all analyzed fragments, 1-min fractions were collected and measured by liquid scintillation counting (as described above).

### Enzyme activity assays.

Radiolabeled PG monomer and dimer fragments purified from size exclusion chromatography of Δ*ldcA* strains were used as a substrate to test 6× His-LdcA activity. Reaction mixtures containing 25 nM (100 ng) of purified LdcA (WT, S165A, E260A, or H331A), 5 × 10^3^ cpm of [^3^H]-labeled GlcNAc-anhMurNAc-tetrapeptide monomer, or 1 × 10^3^ cpm of [^3^H]-labeled peptide-linked dimer and 10 mM Tris-HCl (pH 8.0) were incubated in a total volume of 100 μl for 2 h at 37°C. Reaction mixtures were boiled for 3 min, and the entire reaction volume was processed for separation of the PG monomers and dimers by the use of reversed-phase HPLC and the programs detailed above. One-minute fractions were collected and measured by liquid scintillation counting. Isolated unlabeled PG sacculi from an *ldcA*_S165A_ *pacA*_H329Q_ strain (JL517, described above) were used as the substrate to test LdcA activity on macromolecular PG. A 50-μl volume of purified sacculi was digested with a 250 nM concentration (~1 μg) of either LdcA or LdcAS165A and with 10 mM Tris-HCl (pH 8.0) in a 200-μl total volume for 16 to 18 h at 37°C. Reactions were terminated by boiling for 10 min prior to addition of 30 μl of 1 mg/ml mutanolysin for an additional 16 to 18 h at 37°C. Reactions were terminated by boiling, and insoluble PG was pelleted at 10,000 × *g* for 10 min, filtered through a 10-MWCO spin column, and separated by rpHPLC as described previously (32). An unlabeled PG sacculus preparation (processed as described above) was used to generate substrate for LdcA reactions on peptide-linked PG dimer. Sacculi were digested with the lytic transglycosylase LtgA and separated by rpHPLC as described previously (32). Fractions containing peptide-linked PG dimers were collected with an automatic fraction collector, evaporated, suspended in HPLC-grade water, and used as the substrate in reactions with purified LdcA (WT; S165A) as performed for radiolabeled fragments. Reactions were terminated by boiling, filtered through a 50-MWCO column, and separated by rpHPLC as described above, and products of interest were analyzed by electrospray ionization-TOF (ESI-TOF) mass spectrometry at the University of Wisconsin—Madison Biotechnology Center Mass Spectrometry/Proteomics Facility, as described previously (32).

### NF-κB reporter assays.

HEK-293 cells overexpressing the human versions of NOD1, NOD2, TLR4, and TLR9 and carrying a secreted embryonic alkaline phosphatase (SEAP) reporter (HEK-Blue cells; InvivoGen) were used to measure NF-κB and AP-1 activation essentially as described previously for hNOD1 (58, 61). In accordance with the manufacturer’s instructions, 180-μl volumes of hNOD1 cells were seeded for assays in 96-well plates at 2.8 × 10^5^ cells/ml, hNOD2 and hTLR4 cells were seeded at 1.4 × 10^5^ cells/ml, and TLR9 cells were seeded at 4.5 × 10^5^ cells/ml. Cells were treated in triplicate wells with either 20-μl volumes of bacterial supernatant or appropriate controls as indicated, including 10 μg/ml TriDAP (hNOD1 agonist), 10 μg/ml muramyl dipeptide (MDP; hNOD2 agonist), 10 μg/ml LPS-EK (lipopolysaccharide from *E. coli* K-12; hTLR4 agonist), 50 μg/ml ODN2006 (hTRL9 agonist), or cGCBL media as a blank. Treatments of HEK-Blue hNOD1 and hTLR9 cells were done in parallel with the parental line HEK-Blue Null1-k cells, and treatments of HEK-Blue hNOD2 and hTLR4 cells were done in parallel with the parental line HEK-Blue Null2 cells. Parental cell lines contained the SEAP reporter but did not overexpress pathogen-associated molecular pattern (PAMP) receptors in order to allow subtraction of background NF-κB activation. For treatment of HEK-Blue cells, three independent cultures of nonpiliated *N. gonorrhoeae* MS11 (WT), Δ*ldcA* (JL539), Δ*ldcA* + *ldcA* (JL540), *ldcA*_S165A_ (JL507), *ldcA*_S165A_ + *ldcA* (JL508), *ldcA*_Δ4−75_ (KH619), and *ldcA*_Δ4−75_ + *ldcA* (KH620) strains were grown in liquid culture (cGCBL) on three separate days, with 1 mM IPTG added to cultures where appropriate. For each growth step, culture supernatants were prepared by removing whole cells first by low-speed centrifugation (3,000 × *g*, 5 min) and then by passing supernatants through a 0.22-μm-pore-size syringe filter. Growth of each strain was determined by total protein accumulation in cells, measured by bicinchoninic acid (BCA) assay (Pierce), and supernatants were normalized to cellular growth using blank growth media (cGCBL) prior to treatment. Each of the three independent growths of the strains described above was tested on three independent passages of the HEK-Blue cells, and averages (± standard deviations) of data from each experiment were combined to produce an overall average (± standard error).

HCT116-Dual human colorectal carcinoma cells (InvivoGen) were also used to measure NF-κB and AP-1 activation via the use of a SEAP reporter identical to that used with the HEK-Blue cells. HCT116-Dual cells express multiple pattern recognition receptors, including NOD1, NOD2, TLR3, TLR5, and RIG-I but not TLR2 or TLR4. In accordance with the manufacturer’s instructions, HCT116-Dual cells were washed with PBS, detached with trypsin, suspended in fresh media, and adjusted to 2.8 × 10^5^ cells/ml, prior to seeding 180-μl volumes in wells of a 96-well plate. *N. gonorrhoeae* strains MS11, JL539, JL540, JL507, and JL508 were grown in cGCBL medium–1 mM IPTG for 3 h. One OD_600_ equivalent of bacteria was then harvested from each culture, pelleted for 30 s at 10,000 × *g*, washed once with tissue culture media, pelleted, and suspended again in 1 ml of tissue culture media. Triplicate wells of HCT116 cells were infected with 20 μl of prepared cell suspension from each strain or with 20 μl of a 1:10 or 1:100 dilution of each inoculum. Triplicate control wells were treated with 100 μg/ml Tri-DAP, 100 μg/ml MDP, 100 μg/ml LPS-EK, 100 ng/ml tumor necrosis alpha (TNF-α), 100 ng/ml interleukin-1β (IL-1β), or cGCBL media (blank). Inocula were serially diluted and plated for CFU determination on GCB agar.

All cells were maintained at 37°C in a 5% CO_2_ atmosphere in Dulbecco’s modified Eagle’s medium (DMEM) with 4.5 g/liter glucose and 2 mM l-glutamine, supplemented with 10% fetal bovine serum (FBS) and 100 μg/ml Normocin. When appropriate for selection (according to manufacturer’s instructions), the medium was supplemented with 100 μg/ml zeocin (all cells) and with 30 μg/ml (hNOD1, hNOD2, hTLR4, and hTLR9) or 10 μg/ml (HCT116-Dual) blasticidin. At the start of each assay, the medium described above was exchanged for DMEM with 4.5 g/liter glucose and 2 mM l-glutamine containing 10% heat-inactivated fetal bovine serum (HI-FBS) and lacking the antibiotics described above (test medium). For all of the above-described experiments, the treated/infected cells were incubated for the times suggested by the manufacturer (6 to 24 h, depending on the cell line), after which 20 μl (30 µl for HCT116-Dual) was removed from each well to a new 96-well assay plate and mixed with 180 μl (170 µl for HCT116-Dual) of QUANTI-Blue medium (InvivoGen) for the detection of alkaline phosphatase. After a 1-h incubation, absorbance was read at 650 nm in a plate reader. For HEK-Blue cells, the *A*_650_ value was subtracted from the background measurement of the matching Null1 cell line. With the resulting *A*_650_ value, data (± standard deviations) from triplicate technical replicates of control wells were averaged and the technical replicate averages (± standard errors of the means) of the data from the three biological replicates (for the supernatant from each strain) were averaged. Data were graphed, and statistical analysis was performed in GraphPad Prism 4.0c.

10.1128/mBio.01464-17.5 FIG S5  LdcA active site mutations and signal sequence truncations eliminate release of human NOD1 agonist by GC. (A to D) HEK-293 cells expressing hNOD1 (A), hNOD2 (B), TLR4 (C), or TLR9 (D) and transfected with a secreted alkaline phosphatase (SEAP) reporter for NF-κB activity (HEK-Blue cells) were exposed to cell-free supernatant from growth of strains with *ldcA*_S165A_ or *ldcA*_Δ4−75_ or complemented mutants (*ldcA*_S165A_ + *ldcA*^*+*^, *ldcA*_Δ4−75_ + *ldcA*^*+*^). Samples were assayed simultaneously with the samples and controls represented in Fig. 6, using the same measurement protocol. Values represent means ± standard errors of averages of results from three independent experiments. (E) HCT116 cells transfected with a secreted alkaline phosphatase (SEAP) reporter for NF-κB activity (HCT116-Dual cells) were infected with ~1 × 10^7^ CFU/ml of pilus^+^ Opa^−^ versions of the following strains: the wild-type strain (MS11), mutant Δ*ldcA*, mutant Δ*ldcA* + *ldcA*^*+*^, mutant *ldcA*_S165A_, and mutant *ldcA*_S165A_ + *ldcA*^*+*^ (representing a 10-fold increase over the level shown in Fig. 6E). Samples were assayed simultaneously with samples and controls in Fig. 6E, using the same measurement protocol. Values displayed are the means ± standard deviations of results from triplicate wells determined in one experiment representative of three independent experiments. (A to E) Significance was determined for bracketed comparisons by unpaired *t* test. *, *P* < 0.05; **, *P* < 0.01; ***, *P* < 0.001; n.s., not significant. Download FIG S5, EPS file, 1.1 MB. Copyright © 2017 Lenz et al. 2017 Lenz et al. This content is distributed under the terms of the Creative Commons Attribution 4.0 International license.
